# Clinical Lectures and Reports

**Published:** 1843-11-11

**Authors:** S. Chamberlain

**Affiliations:** Resident Physician


### CLINICAL LECTURES AND REPORTS.

### PHILADELPHIA HOSPITAL.

Service of W. W. Gerhard, M. D.

[Reported by S. Chamberlain, M. D., Resident Physician.]

Case I.—*Tubercular Peritonitis—Arachnitis—Pulmonic Tubercles—Pleuritis—Acute Peritonitis—Death—Autopsy.*

Joseph Stokes, æt. 44, mulatto, was seized about the 20th of June, while at work upon the wharves of the Schuylkill, with a severe sharp pain in the head, which was very soon transferred to the abdomen, and was followed by a gradual enlargement of the belly; the pain continuing, but with less intensity. He entered the hospital on the 27th, with the abdomen greatly distended, suffering at that time little pain, but with a pulse extremely feeble, and strength almost exhausted.

Stimulants, tonics, and a good diet renewed his strength, while revulsives, applied to the abdomen, greatly reduced its size.

Tubercular peritonitis was diagnosticated by Dr. Clymer.* On the 3d of August notes were first taken upon the case.

There was an anxious expression of countenance, and a constantly contracted brow, but no symptoms that were referred to the brain.

Tongue white; bowels rather costive, were moved daily by oil.

Tenderness on pressure at the lower part of the abdomen; meteorism and fluctuation.

The skin was dry and harsh; pulse 70, small and weak. A slight cough had been removed by anodyne mixture; the chest was examined, and no physical signs of disease detected.

August 21. Has had no change of symptoms, excepting occasional pain in the abdomen; relieved at first by hop poultices, and afterwards by flying blisters; and a slight diarrhœa, relieved by oil mixture.

Yesterday he suffered very much with severe pain in the back of his head, which was removed by a single cut-cup.

Aug. 23. Effusion has taken place into the right pleura, producing dyspnœa and great feebleness; pulse scarcely perceptible at the wrist; brandy prescribed.

Aug. 31. A slight diarrhœa again took place, with considerable reduction of the fluid both in pleura and in peritoneum.

September 16. Has suffered more pain in the abdomen, and in increase of fluid in both cavities. To-day diarrhœa has again taken place spontaneously, corresponding with a decrease of fluid as before.

---

* During the temporary absence of Dr. Gerhard in the summer, Dr. Clymer had charge of his service.

Sept. 18. Was aroused from sleep by a sudden sharp pain through his head. Has excessive dyspnœa; respiration is costo-abdominal—cannot be counted; pulse 140, scarcely perceptible at the wrist; face and hands covered with a cold, clammy sweat; fluid in abdomen not more than yesterday—in pleura is very much less.

*Physical Signs.*—Percussion clear at summit, and at middle portion, dull at base; auscultation vesicular at summit, rude at middle, with crepitus at base; posteriorly bronchial at root, crepitous at base; friction sound on the left side; percussion clear; respiration puerile. After a few moments the sounds in the right lung were all obscured by a loud sibilant ronchus. Brandy and carbonate of ammonia were prescribed, but he died in a few hours after visit.

*Post Mortem* 18 *hours after death.*

*Brain.*—Substance natural. Arachnoid of a milky appearance, and somewhat injected; small quantity of yellow serum in the cavity of arachnoid.

*Chest—Right lung.*—Bloody serum in pleura. Numerous adhesions, both old and recent. Pleura rough and thickened, and covered by a false membrane. In the costal pleura, very bright red injection. A few scattered tubercles through the substance of the lung, and hepatization at the base, where it adhered to the diaphragm.

*Left lung.*—Straw-coloured serum. Pleura injected and thickened, but without false membrane. A small mass of tubercles at the summit, and miliary tubercles scattered throughout.

*Liver, Spleen, and Kidnies* healthy, except their peritoneal surface. The whole peritoneum greatly thickened, studded with tubercles, and both surfaces covered with a thick tuberculous false membrane; that over the small intestines completely concealed them, and was itself covered with shreds of more recently effused lymph. No portion of serous membrane was natural. The omentum was but one mass of tubercular matter, a quarter of an inch thick, and presented the appearance of an enlarged pancreas. Small intestines were completely agglutinated by lymph, and presented recent inflammation, as in the pleura.

CASE II.—*Tubercular Peritonitis—Phthisis—Arachnitis—Pleuritis—Acute Peritonitis—Hypertrophy of Left Ventricle—Death—Autopsy.*

John Johnson, æt. 27, mulatto, insane from three years solitary confinement, was treated for pneumonia in February last, and returned to the Lunatic Asylum, apparently cured. Has emaciated considerably since that time. For several weeks past has had a sharp, ringing cough, with but little expectoration.

Entered wards September 13th. Cries and moans very much, as if from pain, but makes no complaint. Respiration is irregular, costo-abdominal, 28 in a minute; cough is constant, short, and often hard and ringing. Dyspnœa often occurs after a severe spell of coughing; sputa usually consists of muco-purulent matter, floating in a serous fluid. Left side of chest is contracted.

*Physical Signs—Right Lung.*—Percussion dull at base, where respiration is also feeble. Left is flat throughout, with bronchial respiration; posteriorly, no vesicular murmur; skin is natural; pulse small and feeble; appetite good; tongue natural and bowels rather loose; abdomen is enlarged, and fluctuation perceptible.

October 7th. The loud ringing cough soon after disappeared, while the sputa became almost entirely purulent. The bronchial sound has increased so far in intensity, that it resembles closely a cavernous ronchus, and a crepitus is heard over a small space posteriorly, where respiration was absent before.

The appetite has been always good, and, at times, excessive. Has had a constant diarrhœa since his entrance, not at any time excessive, and never distressing to him; while the abdomen has continued to increase constantly in size, and fluctuation has become more evident. The pulse has continued feeble, the general strength being little diminished, and emaciation not progressing very much. Skin is soft and of moderate temperature. Has had slight fever, with sweating occasionally in the evening, but no marked hectic.

Oct. 16th. Symptoms have not changed. The enlargement of the abdomen has progressed steadily, notwithstanding diuretics, *calomel*, *squills*, and *digitalis*, &c., and flying blisters to the abdomen. To-day he began to talk more constantly and more wildly than usual, and became extremely restless. Excessive dyspnœa came on, and soon terminated the case.

*Post Mortem* 36 *hours after death.*

*Brain.*—Substance natural; fluid (small quantity) in the ventricles; slight adhesions at the base of the brain; arachnoid of a milky appearance; pia mater congested.

*Chest.*—*Right pleura* contained a large quantity of straw-coloured serum, and was covered (both costal and pulmonary) by a false membrane, rough to the touch, and which was readily stripped off in layers. Scattered solitary tubercles (of a minute size) were found in the pleura. The upper lobe of the lung contained a mass of softened tubercles, surrounded by hepatization; and the third lobe a smaller mass, while the middle one was healthy.

*Left lung* was very much contracted, and adherent to the walls of the chest, very strongly at the summit, and by infiltrated cellular tissue elsewhere. Cavity of the pleura contained very little fluid. Upper lobe of the lung was carnified, and pitted with white encysted tubercular matter, not softened. Similar masses were found between the pleura and pericardium, and between the pleura and diaphragm. No scattered tubercles in the lung, which was pale, and contained air in very small quantity, sufficient to float it, but not to produce crepitation. A strong, tuberculous false membrane, much thicker than on right lung, covered the pleura to the thickness of two lines, and could be stripped off with difficulty, leaving the pleura thin and shrivelled.

*Heart.*—Left ventricle hypertrophied; right thinned and dilated. Serous membrane, both external and internal, was of a milky appearance.

*Liver* enlarged to nearly twice its natural size; seems natural.

*Peritoneum.*—Both surfaces covered by a tuberculous false membrane, two lines in thickness on the parietal portion. Intestines agglutinated, and, at the umbilicus, strongly adherent to the walls of the abdomen; slight and more recent adhesions to the brim of the pelvis. Peritoneal surface of the intestines was studded with miliary tubercles, and presented a limited recent inflammation. The omentum was filled with the tuberculous matter; a quarter of an inch in thickness, as in the case of Stokes.

[Chronic peritonitis occurs only in tuberculous patients. Dr. Louis did not find the anatomical characters of this affection present once out of 300 individuals dead of other chronic diseases besides phthisis. This affection is more common than is generally supposed, and no one is less understood, both in this country and Great Britain. The notorious case of Lady Flora Hastings, one of the ladies of honour to Queen Victoria, which occurred a few years since in London, will no doubt be recollected by our readers. Pregnancy here was first suspected, and much scandal was caused by a wrong diagnosis; the true nature of the disease never seemed to be suspected, until it was revealed at the autopsy.

The symptoms of tubercular peritonitis are generally mild, not numerous, and frequently are unnoticed. They are, however, quite sufficient to warrant a positive diagnosis. The invasion of this disease has no fixed relation with the main affection, and may occur at any period of its progress. The first symptom of chronic peritonitis is enlargement of the abdomen, denoted by the uneasiness in his clothes which the patient experiences, if up and about. With this there is dull abdominal pain, sometimes general, (either singly or co-existent,) increased by pressure and percussion, and unaccompanied by diarrhœa. Later, and at a very variable period, physical exploration, however, reveals fluctuation, and meteorism, to a greater or less degree, will be ascertained. After increasing to a certain extent, fluctuation will diminish, whilst the gaseous distension persists. Where abdominal meteorism is present from the commencement of the disease, without appreciable effusion, it will diminish after some time, leaving the abdomen firmly resistant under pressure, knotted, and the intestinal convolutions marked on the surface; and this tense elasticity persists with complete relaxation of the muscular parietes. Nausea and vomiting rarely occur, unless acute peritonitis should supervene. There is great diversity in the intensity of the rational symptoms; sometimes the feeling of uneasiness, or even positive suffering, is so great as to occupy constantly the attention of the patient; at other times there is entire absence of pain, and the physical signs alone announce the existence of the affection. Ordinarily, the symptoms just enumerated persist, or increase, till death occurs; but sometimes, when the disease is intensely chronic, the abdominal symptoms disappear, and the thoracic symptoms only attract notice. This happened in the the first case. Sometimes abdominal effusion is absorbed with great rapidity, and, on examination after death, you find only general intestinal adhesion by recent false membrane, when, but a few days before, there was positive evidence of fluctuation.

The symptoms of chronic peritonitis, though few, are of great value, from their constancy and regularity, in reference to diagnosis. Dr. Louis places the greatest confidence in them, and states (Recherches sur la Phthisie, 1843, p. 272) that through them he has been led to the successful diagnosis of phthisis in persons in whom there was little or no cough, and where the most careful physical exploration gave no evidence of thoracic disease. The case of Stokes is one of this kind.

To resume, we may say, that where we have general abdominal pain, of moderate intensity, but still sufficiently annoying, without diarrhœa, accompanied with increase in the size and sonorousness of the abdomen, with the supervention of manifest fluctuation, and independent of any organic affection of the viscera, as the liver especially, kidneys, or heart, with subsequent absorption of the effused fluid, leaving a slight, but general, gaseous distension, with the intestinal convolutions well defined, and with this, great loss of strength, which cannot be referred to the condition of the lungs or of the excretions,—when, we say, we have this combination of symptoms, we may, with great safety, diagnosticate tubercular peritonitis.

Tubercular peritonitis may be confounded with cancerous peritonitis. The different epochs of life at which, in general, the two affections occur, the co-existence of cancer in some other organ, the great rarity of cancer in tuberculous patients, with other differential symptoms, will usually serve to distinguish the two diseases.

In the Dublin Journal, for March, 1843, there is a paper on "Strumous Peritonitis with Effusion," by Sir Henry Marsh, which is an evident attempt to describe the disease in question, and is additional evidence of the very scanty knowledge which British practitioners possess of this affection. Some remarks by Dr. Fleetwood Churchill are appended to the communication, in which he agrees with Sir Henry Marsh on the propriety of the principal reliance in the treatment being on mercury; a conclusion which the evidence so far collected on this subject certainly does not warrant. M. C.]

